# The effects of Tai Chi Chuan combined with unstable resistance training on knee muscle strength and dynamic balance in female college students: a randomized controlled study protocol

**DOI:** 10.3389/fpubh.2026.1868830

**Published:** 2026-05-29

**Authors:** Jiping He, Xiaoyun Su, Lin Wang, Jie Men, Qinyuan Yu

**Affiliations:** 1 Department of Rehabilitation Medicine, Shanxi University of Medicine, Fenyang, Shanxi, China; 2 Faculty of Health and Wellness, City University of Macau, Macau, China; 3 Department of Nursing, Shanxi University of Medicine, Fenyang, Shanxi, China; 4 Department of Basic Medicine, Shanxi University of Medicine, Fenyang, Shanxi, China; 5 Department of Rehabilitation Engineering, Anting Hospital, Jiading District, Shanghai, China

**Keywords:** dynamic balance, female college students, knee muscle strength, randomized controlled trial, study protocol, Tai Chi Chuan, unstable resistance training

## Abstract

**Background:**

Insufficient physical activity and prolonged sedentary behavior among college students are associated with reduced lower-limb muscle strength, impaired dynamic balance, and increased susceptibility to sports-related knee injury. Female college students may be especially vulnerable because of sex-specific neuromuscular and biomechanical characteristics. Tai Chi Chuan may improve proprioceptive control and coordinated knee muscle activation, whereas unstable resistance training may rapidly enhance strength and neuromuscular stabilization. Whether a combined programme can produce complementary central-peripheral benefits remains to be verified in a rigorously designed randomized trial.

**Methods:**

This protocol describes a single-center, parallel-group, randomized controlled trial. Fifty-seven healthy female college students aged 18–22 years will be recruited from Fenyang College, Shanxi Medical University, and randomly allocated in a 1:1:1 ratio to a Tai Chi Chuan group (TCC), unstable resistance training group (URT), or Tai Chi Chuan combined with unstable resistance training group (T + URT). All interventions will last 8 weeks, with three 60-min sessions per week. The primary outcomes will be dominant-leg knee extensor peak torque, knee flexor peak torque, and hamstring-to-quadriceps ratio measured using an isokinetic strength testing system at 60 degrees/s. Secondary outcomes will include overall stability index, anteroposterior stability index, and mediolateral stability index under eyes-open and eyes-closed conditions measured with the Biodex Balance System. Assessments will be conducted at baseline and after the 8-week intervention. Intention-to-treat and per-protocol analyses will be used. Missing data will be handled using multiple imputation where appropriate.

**Discussion:**

This trial is designed to determine whether Tai Chi Chuan combined with unstable resistance training provides superior improvements in knee muscle function and dynamic balance compared with either intervention alone.

**Trial registration:**

https://itmctr.ccebtcm.org.cn, Identifier (ITMCTR2026000456).

## Introduction

The decline in physical fitness among university students has become an important public health concern. Reduced skeletal muscle strength, impaired endurance, and diminished balance control are frequently associated with insufficient physical activity and prolonged sedentary behavior (1–3). These changes are particularly relevant in female college students, in whom lower-limb muscle strength and dynamic balance are essential for daily mobility, safe participation in sport, and prevention of knee injury during rapid postural transitions (4, 5). Thus, improving these functional parameters is a critical intermediate target toward lowering the actual incidence of knee injuries in this sedentary population.

Female students may face a higher risk of lower-limb functional impairment because of biomechanical and neuromuscular characteristics that influence knee loading, postural control, and injury susceptibility. Weakness or imbalance between the knee extensors and flexors can compromise joint stability. The hamstring-to-quadriceps ratio (H/Q) is commonly used to reflect the balance between the agonist and antagonist muscle groups around the knee. A more balanced H/Q ratio may contribute to better joint control during movement and may reduce the risk of excessive anterior tibial translation and related knee injuries (6–8).

Dynamic balance is another key determinant of lower-limb functional performance. It requires the integration of visual, vestibular, proprioceptive, and neuromuscular inputs. Under eyes-open conditions, balance performance depends partly on visual feedback, whereas eyes-closed conditions place greater demand on proprioceptive and vestibular control. Exercise strategies that simultaneously improve strength and proprioceptive integration are therefore likely to produce stronger functional benefits than strategies targeting only one component (9–11).

Tai Chi Chuan is a traditional mind–body exercise characterized by slow continuous movements, controlled breathing, alternating weight shifting, semi-squat postures, and sustained attentional regulation. These features may activate the muscles surrounding the knee and enhance proprioceptive awareness. The 24-form simplified Tai Chi Chuan routine is widely used in community and university settings because it is structured, feasible, and safe. However, Tai Chi Chuan is generally a low-to-moderate intensity training modality, and substantial improvements in absolute muscle strength may require prolonged practice (12–14).

Unstable resistance training is a strength-oriented modality performed under unstable surface conditions, such as balance pads or wobble boards. By requiring participants to maintain postural control while performing resisted or bodyweight movements, this training may activate deep stabilizing muscles, improve neuromuscular control, and increase lower-limb strength (15). Compared with stable resistance training, unstable resistance training may be especially relevant for balance-related outcomes (16). Nevertheless, resistance exercises based mainly on squats, lunges, and heel raises may preferentially stimulate the extensor chain and may not fully correct flexor-extensor imbalance (17).

Despite increasing research on Tai Chi Chuan and unstable resistance training, critical knowledge gaps remain: most studies focus on single interventions rather than their combined synergistic effects (18, 19); related work has explored Tai Chi Chuan with instability or core training, but to our knowledge, no three-arm RCT have examined Tai Chi Chuan combined with unstable resistance training in young sedentary female college students; existing evidence is largely based on female athletes or older adults with distinct neuromuscular and fitness profiles (20); and few head-to-head comparisons between single and combined interventions exist to guide optimal exercise prescription. Therefore, this combined intervention represents a novel complementary model: Tai Chi Chuan improves central sensory integration, proprioception and agonist–antagonist coordination, while unstable resistance training enhances peripheral muscle stimulation and strength gains, acting via a central–peripheral mechanism. This randomized controlled trial protocol was designed to fill these gaps and test this hypothesis (18, 20).

## Objectives and hypotheses

The primary objective is to evaluate the effects of an 8-week Tai Chi Chuan combined with unstable resistance training programme on knee flexor and extensor strength in female college students, compared with Tai Chi Chuan alone and unstable resistance training alone.

The secondary objective is to compare the effects of the three intervention modalities on dynamic balance capacity under eyes-open and eyes-closed conditions.

The main hypothesis is that the combined intervention will produce greater improvements in knee flexor peak torque, knee extensor peak torque, H/Q ratio, and dynamic balance indices than either single-modality intervention. A secondary hypothesis is that Tai Chi Chuan alone will show particular advantages in proprioception-dependent balance and H/Q optimization, while unstable resistance training alone will show particular advantages in rapid improvement of extensor strength.

## Methods

### Study design and setting

This is a single-center, parallel-group, randomized controlled study protocol. Participants will be recruited from female undergraduates enrolled at Fenyang College, Shanxi Medical University, China. Eligible participants will be randomly allocated into one of three groups: Tai Chi Chuan group (TCC), unstable resistance training group (URT), or Tai Chi Chuan combined with unstable resistance training group (T + URT).

**Figure 1:**
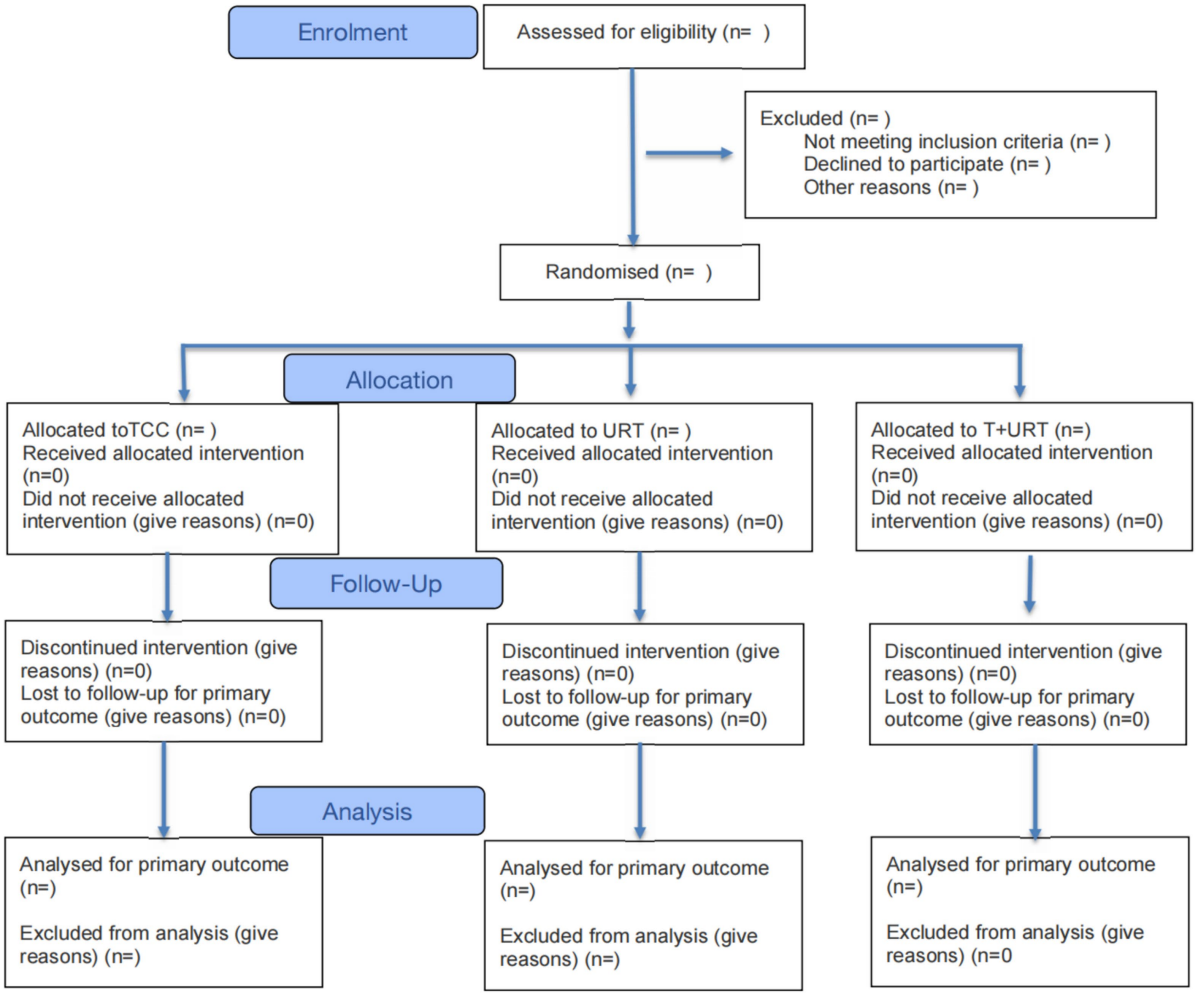
CONSORT 2025 flow diagram.

The trial will follow the principles of the Declaration of Helsinki and the Standard Protocol Items: Recommendations for Interventional Trials (SPIRIT). The study will be conducted in the university training venue and laboratory assessment rooms. All outcome measurements will be performed by trained assessors who are blinded to group allocation. Figure 1 displays a flow diag.

### Participants

Healthy female college students will be recruited through the optional martial arts course and university announcements. Interested students will undergo eligibility screening and will provide written informed consent before enrolment (Table 1). Participants will be asked to maintain their usual diet and lifestyle during the study and to avoid initiating additional structured exercise programmes outside the assigned intervention.

**Table 1:** Eligibility criteria for study participants.

Eligibility domain	Criteria
Inclusion criteria	Female college students aged 18–22 years; normal physical examination; no history of heart, brain, or kidney disease; no recent sports injury; no regular exercise in the previous 3 months; ability and willingness to complete all tests and training; written informed consent; agreement to maintain usual diet and lifestyle.
Exclusion criteria	Abnormal body morphology or obvious limb defects that may affect testing; fracture, muscle injury, or comparable injury within the past 6 months; inability to complete the intervention because of leave, internship, transfer, or similar objective reasons; inadequate training attendance; poor compliance; any adverse condition judged by investigators to increase exercise risk.
Withdrawal criteria	(R1 Q4)Voluntary withdrawal; repeated non-attendance (defined as missing two consecutive training sessions or three cumulative training sessions); development of a condition that makes further participation unsafe (defined as the occurrence of acute exercise-related injury, severe joint pain, cardiovascular discomfort, dizziness, vomiting, or any other health problem judged by the investigator to preclude safe continued training); investigator decision based on participant safety.

### Randomization and allocation concealment

After baseline assessment, eligible participants will be randomly assigned to three groups (Tai Chi Chuan group (TCC), unstable resistance training group (URT), or Tai Chi Chuan combined with unstable resistance training group (T + URT)) in a 1:1:1 ratio using simple randomization. The randomization sequence will be generated by an independent statistician using SPSS (version 25.0, IBM Corp., Armonk, NY, USA). Allocation details will be sealed in sequentially numbered, opaque envelopes. A research assistant not involved in outcome assessment will open the envelopes after baseline testing, just before the first intervention session.

### Blinding

Because of the clear operational differences among the exercise interventions, participants and instructors cannot be blinded. However, outcome assessors, data entry personnel, and statistical analysts will be blinded to group allocation. Group codes will be retained until the main statistical analysis has been completed. This approach is intended to reduce measurement and analysis bias while preserving the feasibility of the intervention.

### Interventions

All groups will train for 8 weeks, three sessions per week, 60 min per session. Each session will include a standardized 10-min warm-up and a standardized 10-min cool-down. Exercise intensity is designed based on the progressive overload, specificity, and neuromuscular adaptation principles of sports coaching, and monitored using a Polar heart rate monitor. Target intensity will gradually increase from approximately 50% of maximum heart rate in week 1 to approximately 60% of maximum heart rate from week 3 onward. There are no differences between the prescribed intensity and the actual training performed, as real-time heart rate monitoring and on-site instructor supervision ensure strict implementation of the intensity protocol. Maximum heart rate will be estimated as 220 minus age. Training instructors will record attendance, heart rate, adverse events, and protocol deviations after each session (Table 2) (21, 22).

**Table 2:** Standardized warm-up, cool-down, intensity monitoring and safety supervision protocol.

Component	Protocol
Warm-up	Deep breathing for 1 min; dynamic stretching of upper limbs and trunk for 3 min; static stretching of lower-limb muscles for 3 min, focusing on quadriceps, hamstrings, and gluteal muscles; knee rotations and ankle flexion-extension for 3 min.
Cool-down	Whole-body static stretching for 6 min followed by slow walking for 4 min.
Intensity monitoring	Heart rate monitored in real time; intensity progressed from 50% HRmax during early adaptation to 60% HRmax during later weeks; HRmax calculated as 220 - age.
Safety supervision	Sessions supervised by trained instructors; movement quality, fatigue, dizziness, pain, and injury signs monitored continuously.

### Tai chi Chuan group

**Figure 2:**
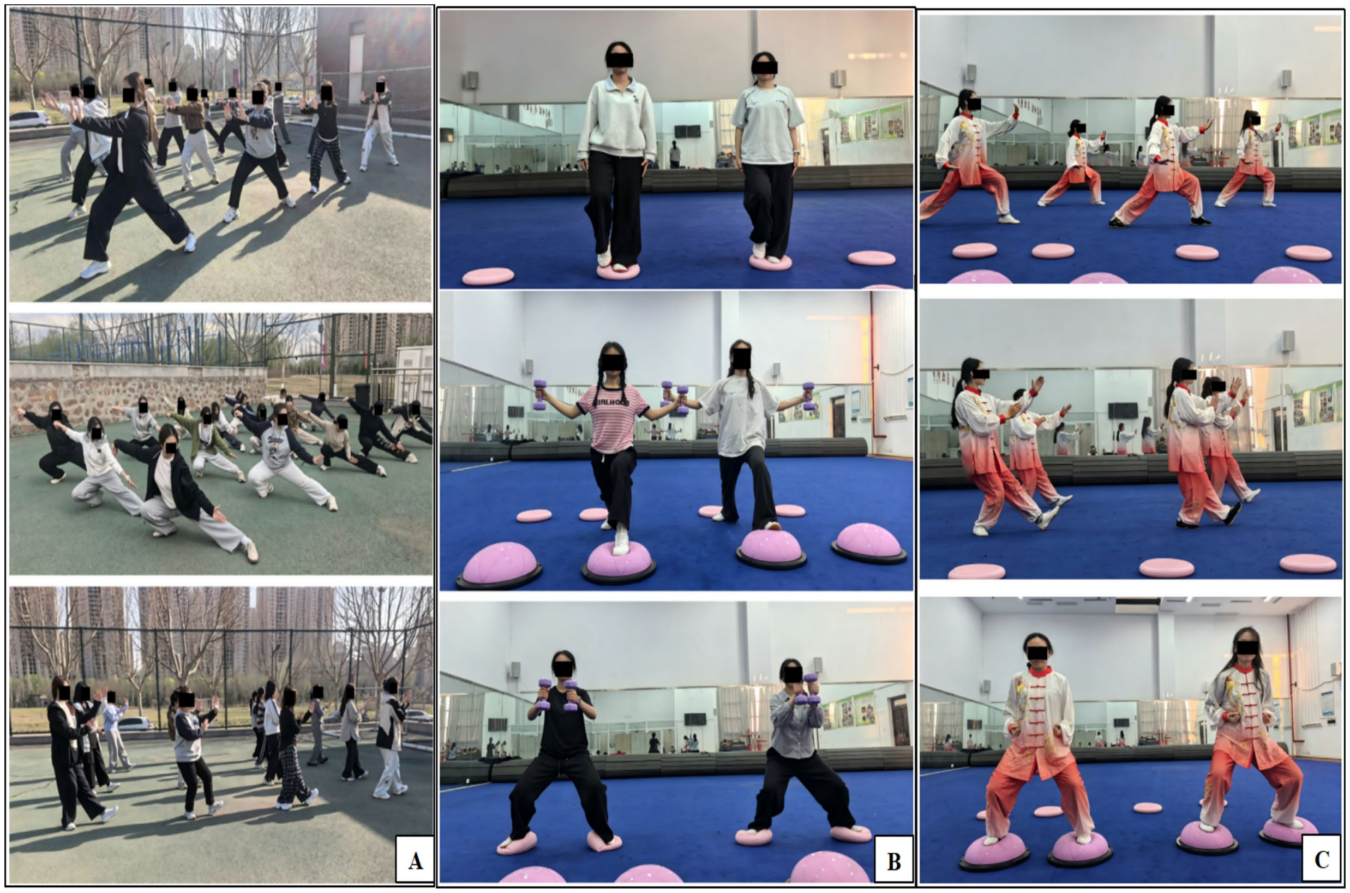
Training images for the three groups. Column A displays training photographs of the TCC group; Column B displays training photographs of the URT group; Column C displays training photographs of the T + URT group.

Participants allocated to the TCC group will receive 24-form simplified Tai Chi Chuan training using a phased learning and training model. All exercises will be performed on stable ground without external resistance equipment (Table 3 and Figure 2). Continuous practice of the 24-form Tai Chi Chuan for approximately 8 min has been shown to maintain steady-state activation of the muscles around the knee (especially the vastus medialis and biceps femoris) and sustained proprioceptive demand, without excessive fatigue that would compromise movement quality (18). Furthermore, the present study adopts a protocol of 7–8 sets per session, with a total training volume of approximately 60 min, which is consistent with effective intervention protocols in young adults (23).

**Table 3:** Phased training protocol for the Tai Chi Chuan group.

Phase	Weeks	Training content	Training requirements
Learning phase	1–2	Explanation of basic Tai Chi Chuan movements, body weight shifting, breathing methods, and segmented learning of the first 12 movements.	One to two movements taught per day; demonstration videos provided after each session; participants required to independently complete segmented movements.
Consolidation phase	3–4	Completion of the full 24-form Tai Chi Chuan routine and repeated practice to correct movement standardization.	Fluid movements, stable body weight distribution, coordinated breathing, and consistent pace of approximately 8 min per set.
Training phase	5–8	Continuous practice of the full 24-form routine, approximately 8 min per set, 7–8 sets per session, with 1-min rest intervals.	Maintain 50–60% HRmax; emphasize proper form, knee muscle activation, and proprioceptive awareness.

### Unstable resistance training group

Participants allocated to the URT group will receive lower-extremity unstable resistance training using balance pads and wobble boards. The protocol will progress from bodyweight to light external resistance, targeting the knee flexor-extensor muscle groups and core stabilizing muscles (Table 4 and Figure 2). According to ACSM guidelines, a 30-s contraction time (approximately 12–15 repetitions) on unstable surfaces is sufficient to induce strength-endurance adaptations without early fatigue-induced postural sway, and a 1:2 work-to-rest ratio (30s work / 60s rest) maintains movement quality and reduces injury risk (24, 25).

**Table 4:** Phased training protocol for the unstable resistance training group.

Phase	Weeks	Training content	Load/rest	Training requirements
Adaptation	1–2	Wall-assisted squats on a balance pad; lunges on a balance pad; static heel raises on a balance pad; single-leg standing on a balance pad.	30 s per set, 3 sets, 60-s rest between sets.	Maintain stable posture; avoid knee hyperextension or abduction; focus on deep muscle activation.
Improvement	3–4	Dynamic half-squats on a balance pad; dynamic lunges on a balance pad; single-leg touch-down exercise on a balance pad; static half-squats on a wobble board.	30 s per set, 3 sets, 60-s rest between sets.	Maintain center-of-gravity control; coordinate slow knee flexion-extension with movement rhythm.
Consolidation	5–8	Static knee squats while holding a medicine ball on a balance pad; weighted reverse lunges on a wobble board with 1–2 kg dumbbells; weighted heel raises on a balance pad with 1–2 kg dumbbells.	20 s static squats per set or 15 repetitions per set; 3 sets per movement; 60-s rest between sets.	Apply light external resistance; maintain standardized movement; avoid excessive swaying; enhance knee strength control.

### Combined intervention group

Participants allocated to the T + URT group will complete 30 min of Tai Chi Chuan followed immediately by 30 min of unstable resistance training during each 60-min session. The first 30 min will include 3–4 sets of the complete 24-form Tai Chi Chuan routine. The subsequent 30 min will include the unstable resistance exercises corresponding to the current phase of the URT protocol. No additional rest interval will be inserted between the Tai Chi Chuan and resistance components. The overall training intensity will be maintained at 50–60% HRmax.

### Outcome measures

All outcome assessments will be completed within 1 week before the intervention and within 1 week after completion of the 8-week intervention. The testing sequence will remain consistent across participants and time points. The dominant leg (preferred kicking leg) will be used for knee muscle strength testing (Table 5).

**Table 5:** Outcome measures, assessment tools and interpretation criteria.

Outcome category	Measure	Instrument	Interpretation
Primary outcome	Knee extensor peak torque (EPT)	Multi-joint isokinetic strength testing and training system, Model A8-2, at 60 degrees/s	Higher value indicates greater knee extensor strength.
Primary outcome	Knee flexor peak torque (FPT)	Multi-joint isokinetic strength testing and training system, Model A8-2, at 60 degrees/s	Higher value indicates greater knee flexor strength.
Primary outcome	Hamstring-to-quadriceps ratio (H/Q)	Calculated from flexor and extensor peak torque	Higher balanced ratio indicates improved flexor-extensor strength balance.
Secondary outcome	Overall Stability Index (SI)	Biodex Balance System, bipedal dynamic balance test	Lower value indicates better balance capacity.
Secondary outcome	Anteroposterior Stability Index (APSI)	Biodex Balance System, bipedal dynamic balance test	Lower value indicates better anterior–posterior stability.
Secondary outcome	Mediolateral Stability Index (MLSI)	Biodex Balance System, bipedal dynamic balance test	Lower value indicates better medial-lateral stability.
Safety outcome	Adverse events and exercise-related discomfort	Instructor record form and participant report	Events summarized by type, severity, relation to intervention, and outcome.

### Knee muscle strength testing

A multi-joint isokinetic strength testing and training system will be used to assess knee muscle strength. Isokinetic testing is the gold standard for evaluating maximal knee strength, and testing at an angular velocity of 60°/s is the most efficient and reliable approach for measuring maximal muscle strength (26). Therefore, peak torque of the knee extensors and flexors, as well as the H/Q ratio, will be measured at 60°/s. An H/Q ratio ≥ 0.60 is considered desirable, whereas a ratio < 0.55 indicates relative hamstring weakness and an increased risk of non-contact knee injury. An increase in the H/Q ratio after the intervention will be interpreted as improved muscle balance (27). Before formal testing, each participant will complete 3–5 submaximal knee flexion-extension repetitions as a warm-up. Under the supervision of a qualified therapist and within a pain-free range of motion, the participant will then perform five maximal-effort knee flexion and extension repetitions to ensure accurate data collection (28). The dominant leg (preferred kicking leg, cross-verified by a functional task such as stepping onto a curb (29)) will be tested. Testing only the dominant leg avoids confounding from baseline strength differences between legs.

### Dynamic balance testing

Dynamic balance will be evaluated using the Biodex Balance System. This device has established good-to-excellent intratester reliability for stability indices in healthy university students (ICC = 0.85 for SI, 0.78 for APSI, and 0.84 for MLSI during static stance) and has been widely validated for dynamic balance assessment (30). Participants will stand barefoot on the platform at stability level 8, with fixed heel coordinates and toes abducted at approximately 30 degrees. During the eyes-open test, participants will focus on the screen and attempt to keep the cursor centered for 20 s. During the eyes-closed test, the same posture and task duration will be used without visual input. Each condition will be repeated three times, and the mean value will be used for analysis. SI, APSI, and MLSI will be extracted. Lower index values indicate better balance capacity (31).

### Sample size

The sample size will be estimated using PASS 15.0 software. Knee extensor peak torque will be selected as the primary outcome for sample size calculation. Based on relevant preliminary and published experimental data, the expected mean and standard deviation of post-intervention knee extensor peak torque in the three groups will be used to estimate an effect size of Cohen’s *f* = 0.55. With a two-sided alpha level of 0.05, statistical power of 0.90, and one-way analysis of variance for three-group comparison, the minimum required sample size is 16 participants per group. Allowing for an anticipated dropout rate of approximately 15%, 19 participants will be enrolled in each group, yielding a total sample size of 57 participants (32).

### Adherence and intervention fidelity

Attendance will be recorded for every session. A participant will be considered adherent if she meets both of the following criteria:

(1) Training frequency adherence: Attendance rate ≥80%; (2) No major protocol deviation: defined as any unapproved change to the intervention content, duration, intensity, or progression that could systematically affect outcome measures, including but not limited to: (a) performing a different Tai Chi form than the 24-form simplified routine, (b) using unstable surfaces or resistance loads outside the prescribed progression in the URT protocol, (c) combining outside structured exercise programmes that interfere with the intervention effect, or (d) persistent incorrect movement patterns that, despite instructor correction, compromise training safety or fidelity. Minor deviations (e.g., occasional missed warm-up exercises, temporary heart rate outside the target zone for <10% of session time) will be recorded but will not automatically classify the participant as non-adherent.

Instructors will follow standardized checklists to ensure intervention fidelity. Heart rate and observations will verify exercise intensity and quality. Missed sessions will be followed up to support continued participation where safe.

### Participant timeline

**Figure 3:**
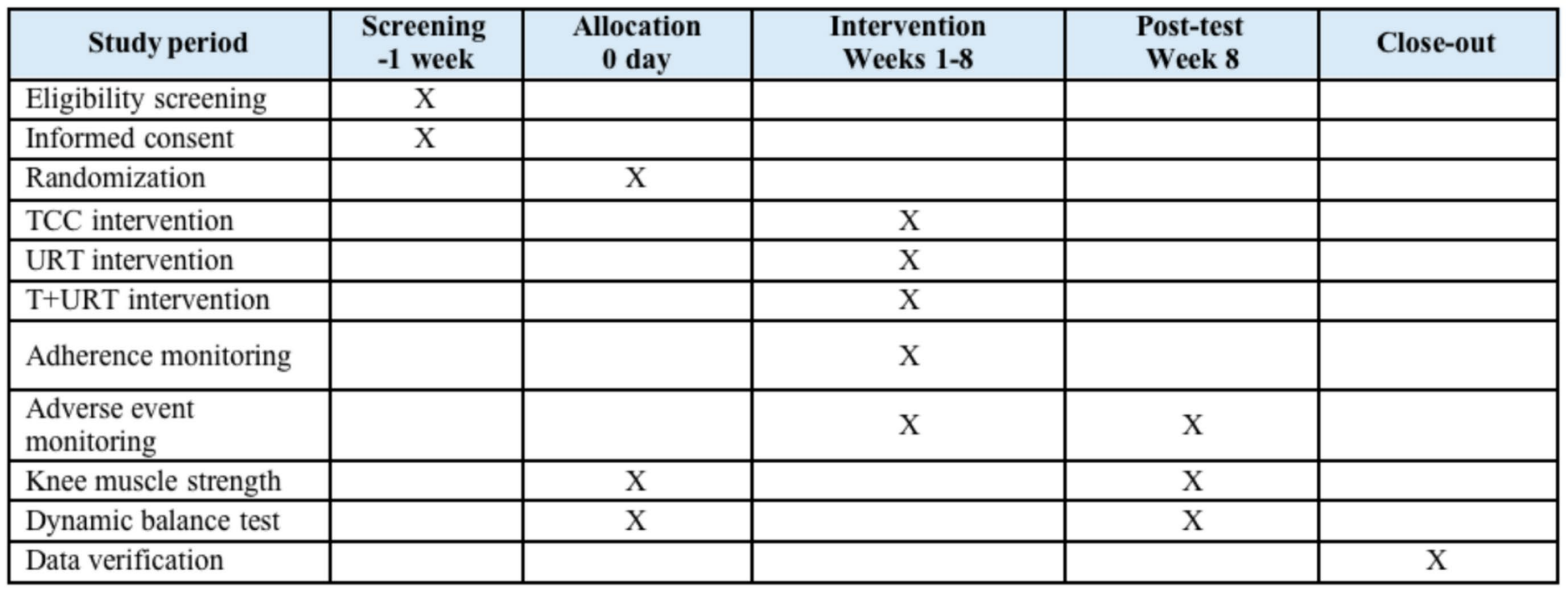
Schedule of enrolment, interventions, and assessments.

All participants are expected to complete the trial within 8 weeks following enrollment. Recruitment and baseline assessments will begin in May 2026, with interventions and post-intervention assessments conducted consecutively. The overall schedule of enrollment, interventions, and participant assessments is illustrated in a temporal schematic diagram (Figure 3).

### Data collection and management

Clinical and training data will be recorded on paper case report forms and then entered into an electronic spreadsheet by trained research staff. Double data entry or independent verification will be used for key variables. All electronic files will be password protected. Paper forms will be stored in locked cabinets accessible only to authorized study personnel. Participant names will be replaced by study identification codes in the analysis dataset. Data will be retained according to institutional requirements.

### Statistical analysis

Two analysis sets will be used. The intention-to-treat set will include all randomized participants in their originally allocated groups regardless of adherence or withdrawal. The per-protocol set will include participants who complete the full intervention protocol without major protocol deviations. The primary analysis will be based on the intention-to-treat principle, and the per-protocol analysis will be used as a sensitivity analysis.

Missing data will be handled using multiple imputation when the missingness mechanism is considered missing at random. Baseline knee muscle strength, dynamic balance index values, age, height, and weight will be considered predictor variables for imputation. Five imputed datasets will be generated using a multiple linear regression model in SPSS 25.0. Pooled estimates will be calculated according to Rubin’s rules. If missing data are minimal, complete-case analysis may be reported as a supplementary analysis.

Categorical variables will be expressed as n (%). Normally distributed quantitative variables will be presented as mean ± standard deviation, and non-normal data as median (interquartile range). Normality will be tested with the Shapiro–Wilk test, and variance homogeneity with Levene’s test. For within-group pre–post comparisons, paired t-tests will be used for normally distributed data; otherwise, Wilcoxon signed-rank tests will be applied. Between-group differences will be assessed using one-way ANOVA for parametric data, followed by Bonferroni-adjusted pairwise comparisons when the overall effect is significant. Non-parametric data will be analyzed with the Kruskal–Wallis test, followed by Dunn–Bonferroni pairwise comparisons. Categorical variables will be compared using the chi-square test or Fisher’s exact test. Given three primary outcomes (knee extensor peak torque, knee flexor peak torque, and hamstring-to-quadriceps ratio), a Bonferroni correction will be applied to control multiplicity, with a significance threshold set at *p* < 0.017 (0.05/3). Secondary outcomes are exploratory, a two-sided *p* < 0.05 will be used without multiplicity correction.

### Safety monitoring

Potential adverse events include muscle soreness, fatigue, dizziness, ankle or knee discomfort, falls, sprains, and other exercise-related injuries. Instructors will monitor participants during every training session and will terminate or modify exercises if participants report pain, dizziness, or excessive fatigue. All adverse events will be documented with onset time, severity, management, relationship to the intervention, and outcome. Serious adverse events will be reported to the ethics committee according to institutional procedures.

### Quality control and data monitoring

A study coordination group will supervise recruitment, training delivery, outcome assessment, data entry, and statistical analysis. Before the start of the trial, all instructors and assessors will receive standardized training on the intervention manual, test procedures, equipment calibration, adverse event reporting, and participant communication. The same training venue, test environment, and equipment settings will be used as far as possible to reduce measurement variability.

The isokinetic testing system and Biodex Balance System will be checked before assessment days according to manufacturer recommendations. Assessors will follow a written operating procedure for participant positioning, verbal encouragement, rest intervals, and data recording. To minimize performance bias, participants will be instructed not to discuss their group allocation with outcome assessors.

The principal investigator will review study documents at regular intervals. Items reviewed will include informed consent forms, screening records, randomization records, attendance sheets, heart rate records, adverse event forms, and original assessment data. Any protocol deviation will be documented, classified as major or minor, and considered in the per-protocol analysis plan.

### Confidentiality and participant protection

All participant information will be handled confidentially. Each participant will receive a unique study identification code, and this code will be used on case report forms and in the analysis database. Identifiable information will be stored separately from outcome data. Only authorized members of the study team will have access to the linkage file. Public reports and publications will present aggregated results without revealing participant identity.

Participants will be informed that participation is voluntary and that withdrawal from the study will not influence their course assessment or access to university services. If discomfort or injury occurs during training or testing, the study team will provide immediate first aid and will assist the participant in obtaining appropriate medical evaluation when needed.

### Protocol amendments

Any important change to the protocol, including changes to eligibility criteria, intervention procedures, outcome measures, sample size, or analysis methods, will be submitted to the ethics committee for review before implementation. Approved amendments will be documented with version number, date, rationale, and affected sections. Investigators, instructors, and assessors will be informed of approved changes before the updated procedures are implemented.

### Ethics and dissemination

This study has been approved by the Scientific Research Ethics Committee of Fenyang College, Shanxi Medical University (Approval No. 2025078). Written informed consent will be obtained before enrolment. Participation will be voluntary, and participants may withdraw at any time without penalty. The study will be conducted in accordance with the Declaration of Helsinki and relevant institutional guidelines. Findings will be disseminated through peer-reviewed publications, conference presentations, and university health promotion materials.

## Discussion

This study protocol proposes a rigorous randomized controlled trial to evaluate whether Tai Chi Chuan combined with unstable resistance training can improve knee muscle strength and dynamic balance in female college students. The study responds to a practical need: many college students have insufficient physical activity, while female students may be at increased risk of lower-limb injury during sport and daily physical activity (7, 33, 34). An efficient, safe, and feasible training model may therefore have value for university health promotion.

The combined intervention is designed around a central-peripheral rationale. Tai Chi Chuan emphasizes movement awareness, controlled weight shifting, breathing coordination, and continuous adjustments of the body center of mass. These characteristics may support central sensory integration and proprioceptive control (35–37). Unstable resistance training, by contrast, provides stronger peripheral loading to the knee musculature and challenges stabilizing muscles through unstable surfaces (38, 39). The combination is expected to improve both the neural control and muscular execution components of lower-limb function.

Compared with Tai Chi Chuan alone, the combined programme may overcome the limitation of slower strength improvement by adding targeted resistance stimulation. Compared with unstable resistance training alone, it may compensate for the limitation of extensor-dominant loading by improving flexor-extensor coordination and proprioceptive regulation. Therefore, the combined intervention may produce broader functional benefits than either single intervention (40, 41).

To our knowledge, no three-arm RCT has directly compared Tai Chi Chuan combined with unstable resistance training versus each intervention alone in sedentary female college students, despite prior work examining Tai Chi with instability, vibration, or core training in other populations or designs (18, 42). Existing Tai Chi studies have largely focused on older adults or clinical populations, with limited data on muscle strength gains in young females (12, 43). Unstable resistance training studies have mainly involved healthy or athletic populations, rarely examining the hamstring-to-quadriceps ratio or eyes-closed dynamic balance (39, 44, 45). This protocol fills this gap by evaluating a central-peripheral complementary approach. Conversely, if no added benefit is found, this would suggest excessive neuromuscular demand without further gain — an equally informative finding. By comparing three arms, this trial will provide the first high-level evidence on whether combining these two training philosophies yields synergistic effects on knee function and dynamic balance in young sedentary women.

This protocol includes several methodological strengths. First, the randomized three-arm design allows direct comparison between two single-modality interventions and their combination. Second, objective laboratory measures will be used for both strength and balance outcomes. Third, blinded outcome assessment and statistical analysis will reduce bias. Fourth, both intention-to-treat and per-protocol analyses will be planned to evaluate robustness. Fifth, intervention progression, heart rate monitoring, and standardized warm-up and cool-down procedures will improve intervention fidelity and safety.

The study also has limitations. The trial will be conducted at a single center and will include only healthy female college students aged 18–22 years, which may limit generalizability to male students, older adults, patients, or athletes. The intervention period will last 8 weeks, and long-term follow-up is not planned in the current protocol; therefore, persistence of training effects will remain uncertain. The study focuses on knee muscle strength and dynamic balance rather than actual injury incidence. In addition, future studies may incorporate advanced evaluation frameworks such as feature fusion and machine learning into outcome assessments to improve the precision of functional ability measurements (46).
